# Live birth after oocyte donation in women with Turner syndrome compared with other causes of premature ovarian insufficiency: a retrospective cohort study

**DOI:** 10.1007/s10815-026-03906-1

**Published:** 2026-05-25

**Authors:** Hugo Saignes, Nicolas Romain-Scelle, Elsa Labrune, Gaëlle Soignon, Laurence Pral-Chatillon, Bruno Salle, Aude Brac de la Perrière, Eloïse Fraison

**Affiliations:** 1 Department of Reproductive Medicine and Biology, Hospices Civils de Lyon, Lyon, France; 2 Claude Bernard University Lyon 1, Villeurbanne, France; 3 UMR, CNRS 5558, Biometry and Evolutionary Biology Laboratory, Villeurbanne, France; 4 U1208, Inserm, Bron, France; 5 Faculté de Médecine Lyon Sud, Oullins, France; 6 Department of Endocrinology, Hospices Civils de Lyon, Lyon, France

**Keywords:** Turner syndrome, Oocyte donation, Primary ovarian insufficiency, Uterine volume, Assisted reproduction

## Abstract

**Purpose:**

To compare live birth rates after in vitro fertilization (IVF) with oocyte donation between women with Turner syndrome and women with premature ovarian insufficiency (POI) of other etiologies and to evaluate the relationships between uterine volume, duration of hormone replacement therapy (HRT), and obstetric outcomes.

**Methods:**

This retrospective cohort study included 97 women with POI undergoing IVF with oocyte donation at a university-affiliated tertiary care center between 2007 and 2023, including 30 women with Turner syndrome and 67 with POI of other etiologies. Artificial endometrial preparation cycles were used for all recipients. Uterine volume was measured by transvaginal ultrasound prior to the first embryo transfer. Primary outcome was live birth per embryo transfer. Secondary outcomes included live birth according to embryo transfer rank, associations between uterine volume and obstetric or neonatal outcomes, and the relationship between uterine volume and duration of prior HRT.

**Results:**

A total of 245 embryo transfers were performed. Live birth rates per embryo transfer were comparable between women with Turner syndrome and those with other POI etiologies (29% vs. 27%). However, women with Turner syndrome had a significantly lower probability of achieving live birth at the first embryo transfer (adjusted odds ratio [OR] 0.09; 95% confidence interval [CI] 0.01–0.64), whereas no significant difference was observed after three or more transfers. Median uterine volume prior to IVF did not differ significantly between groups, despite a substantially longer duration of prior HRT in women with Turner syndrome (15.5 vs. 3.0 years). No significant association was observed between uterine volume and duration of HRT in either group. Among singleton pregnancies, decreasing uterine volume was associated with increased risks of preeclampsia, intrauterine growth restriction, non-cephalic presentation, and adverse neonatal outcomes.

**Conclusions:**

Women with Turner syndrome achieve live birth rates after IVF with oocyte donation comparable to those of women with other POI etiologies, although more embryo transfer attempts may be required. No clear association between uterine volume and duration of HRT was observed. However, decreasing uterine volume was associated with adverse obstetric outcomes, highlighting the importance of early hormonal management and careful obstetric surveillance in this population.

## Introduction

Turner syndrome is one of the most common chromosomal abnormalities and represents the most frequent sex chromosome abnormality in females, occurring in approximately 1 in 2500 female live births. It results from the complete or partial absence of one X chromosome and is classically classified into complete (45,X) and mosaic forms [1]. More than 95% of individuals with Turner syndrome exhibit premature ovarian insufficiency (POI), defined as the cessation of ovarian function before the age of 40. Only about 5% of women with Turner syndrome can conceive spontaneously, and those pregnancies are associated with high rates of early miscarriage [2].

As a result, most women with Turner syndrome are infertile and require in vitro fertilization (IVF) with oocyte donation to achieve pregnancy. Existing literature suggests that clinical pregnancy rates after oocyte donation are comparable between women with Turner-related POI and those with POI of other etiologies and normal karyotypes [3]. However, women with Turner syndrome appear to experience a higher incidence of early pregnancy loss [4]. Several hypotheses have been proposed to explain this discrepancy, including impaired endometrial receptivity due to X-chromosome haploinsufficiency, reduced uterine volume with thinner endometrial lining, and a possible deleterious effect of autoimmunity [5], which is more prevalent in Turner syndrome [6].

One of the key determinants of uterine development in women with POI is the duration of hormone replacement therapy (HRT) administered prior to attempting pregnancy [7]. In addition, pregnancies obtained through oocyte donation in this population are associated with an increased risk of obstetric complications, including hypertensive disorders and fetal growth restriction [8].

The primary objective of this study was to compare live birth rates after IVF with oocyte donation between women with Turner syndrome and those with POI of other etiologies.

The secondary objectives were to evaluate the correlation between uterine volume and the duration of prior HRT and the association between pre-treatment uterine volume and obstetric outcomes.

## Material and methods

### Study design and participant

This was a retrospective, single-center cohort study conducted in the Department of Reproductive Medicine at the Hôpital Femme Mère Enfant, Hospices Civils de Lyon, France.

We included all women aged 18 to 40 years who underwent at least one embryo transfer following IVF with oocyte donation for the indication of POI, with or without Turner syndrome, between January 1, 2007, and December 31, 2023.

Turner syndrome was defined by karyotype analysis showing either complete monosomy X (45,X) or mosaicism with at least 5% of 45,X cell lines.

We excluded women who had undergone total body irradiation and/or pelvic radiotherapy.

All women with Turner syndrome underwent a comprehensive preconception multidisciplinary evaluation prior to approval for assisted reproductive technology (ART). This assessment systematically included cardiovascular screening with measurement of blood pressure, evaluation of aortic diameter indexed to body surface area using echocardiography or cardiac MRI, and electrocardiogram. Contraindications to pregnancy were assessed according to national guidelines [9]. Absolute contraindications included a history of aortic dissection or aortic valve replacement, as well as an indexed ascending aortic diameter > 25 mm/m^2^, or > 20 mm/m^2^ in the presence of risk factors such as bicuspid aortic valve, coarctation of the aorta, or transverse arch elongation. Uncontrolled hypertension was considered a temporary contraindication.

### Ovarian stimulation and oocyte donation protocol

All oocyte donors met the requirements stipulated by French law. Donors underwent pretreatment with a combined oral contraceptive pill followed by a 5-day wash-out period before controlled ovarian stimulation. Stimulation was performed using recombinant follicle-stimulating hormone (FSH) in an antagonist protocol, with the addition of ganirelix starting on stimulation day 6. Final oocyte maturation was triggered with a gonadotropin-releasing hormone (GnRH) agonist (triptorelin).

Oocytes were retrieved by transvaginal aspiration under general anesthesia in an operating theater. Recovered oocytes were either vitrified after denudation or used fresh, depending on the clinical context.

All couples underwent a complete semen analysis, including sperm count, morphology assessment, sperm culture, and motile sperm count, and all results were compatible with the use of intracytoplasmic sperm injection (ICSI). No severe male factor infertility was identified in this cohort.

### Endometrial preparation and embryo transfer

All recipient women with POI included in the study received HRT to prepare an artificial endometrial cycle. Estradiol was administered either vaginally in a step-up regimen (4 mg, then 6 mg, then 8 mg daily) or at a constant dose (8 mg daily), or via transdermal patches at a constant dose of 150 µg, replaced every 3 days. Serial transvaginal ultrasounds were performed to monitor endometrial thickness and uterine artery pulsatility index. Monitoring continued until an endometrial thickness ≥ 7 mm and a mean uterine artery pulsatility index < 3 were achieved.

Progesterone supplementation was then initiated, either exclusively via the vaginal route (600 mg micronized progesterone daily) or using a combined regimen of vaginal progesterone and oral dydrogesterone (30 mg daily). The duration of progesterone exposure prior to embryo transfer was adapted to the developmental stage of the embryos.

Preimplantation genetic testing for aneuploidy (PGT-A) was not performed in this cohort, in accordance with French regulations.

Fresh or frozen embryos were transferred into the uterine cavity, using a soft catheter, by a senior physician. All cleaved embryos exhibited a fragmentation rate below 30%, and blastocysts were graded as AA, AB, or BA according to the Gardner classification [10].

### Data collection

Patient and embryo’s transfer characteristics were collected retrospectively from medical records.

Baseline uterine ultrasound measurements prior to the first embryo transfer were recorded. Pelvic ultrasounds were performed transvaginally, and uterine biometric parameters—including uterine length (L), anteroposterior diameter (AP), and width (l)—were measured, including the cervical component. Uterine volume was calculated using the ellipsoid model formula: L × l × AP × 0.52 [11].

Uterine factors potentially affecting uterine size or fertility were systematically assessed during this ultrasound prior to ART, including the presence of uterine fibroids, adenomyosis, congenital uterine anomalies, and endometrial polyps.

Obstetrical and neonatal outcomes were analyzed in singleton pregnancies only, with exclusion of twin pregnancies to avoid bias related to the higher morbidity associated with multiple gestations.

### Statistical analysis

Continuous variables were described as median (interquartile range), and categorical variables were expressed as counts (percentages). Univariate comparisons between groups were performed using standardized mean differences (SMD) with corresponding 95% confidence intervals.

Embryo transfer success, defined as the achievement of a live birth, according to Turner syndrome status, was analyzed using an adjusted logistic regression model. Odds ratios (OR) were estimated stratified on the rank of embryo transfer attempts for each patient.

Each OR was adjusted for body mass index (BMI), history of spontaneous puberty, type of embryo transferred (fresh or warmed), day of embryo development at transfer, number of embryos transferred, type of artificial cycle used for endometrial preparation, duration of estradiol administration as monotherapy, and endometrial thickness prior to embryo transfer.

The association between uterine volume and obstetrical outcomes was conducted using a logistic regression model stratified by Turner status without adjustment variables and estimated using ORs (expressed for a 1 mL reduction). Only singleton pregnancies were analyzed (twin pregnancies were excluded from this analysis because of their own morbidity).

The association between duration of HRT and uterine volume was conducted using a linear regression model stratified by Turner status without adjustment variables and estimated using mean volume change per unit HRT duration change.

### Ethics

This study was approved by the local ethics committee (Agora n°23–5468).

## Results

### Study population

Between January 2007 and December 2023, a total of 97 women with POI who underwent IVF with oocyte donation were included, comprising 30 women with Turner syndrome and 67 women with POI of other etiologies. Overall, 245 embryo transfers were performed during the study period.

### Patient characteristics

Baseline characteristics of the study population are summarized in Table 1.

**Table 1:** Patient characteristics

Main characteristics	*N*	Overall	Turner *N* = 30	Other POI^a^ *N* = 67	Difference (G5% CI^b^)
Age (years), median (IǪR^c^)	97	32 (30–35)	31 (30–32)	32 (30–35)	0.25 (− 0.18 to 0.69)
Height (cm), median (IǪR^c^)		162 (156–167)	155 (152–158)	165 (160–170)	0.28 (− 0.15 to 0.71)
BMI^e^ (kg/m^2^), median (IǪR^c^)		22.2 (20.5–25.2)	23.1 (21.7–28.9)	21.6 (19.8–24.8)	− 0.49 (− 0.92 to − 0.05)
Karyotype					
45,X, *n* (%)	97	20 (21)	20 (67)	-	
Mosaic, *n* (%)		10 (10)	10 (33)	-	0.50 (0.06 to 0.94)
46,XX, *n* (%)		67 (69)	-	67 (100)	
Endocrine follow-up					
Spontaneous puberty, *n* (%)	95	65 (68)	10 (36)	55 (82)	1.10 (0.60 to 1.50)
Prior HRT^d^ duration (years), median (IǪR^c^)	76	4.0 (2.0 − 13.5)	15.5 (12.0 − 17.0)	3.0 (2.0 − 6.0)	− 1.50 (− 2.10 to − 1.00)
Uterine volume (mL), median (IǪR^c^)	63	38 (22 − 51)	44 (25 − 51)	32 (21 − 48)	− 0.20 (− 0.72 to 0.32)
Medical history					
Gravidity, *n* (%)	96	0.00 (0.00–0.00)	0.00 (0.00–0.00)	0.00 (0.00–0.00)	0.36 (− 0.08 to 0.79)
Parity, *n* (%)		0.00 (0.00–0.00)	0.00 (0.00–0.00)	0.00 (0.00–0.00)	0.31 (− 0.13 to 0.74)
Diabetes mellitus, *n* (%)		2 (2.1)	1 (3.4)	1 (1.5)	0.13 (− 0.31 to 0.56)
Hypothyroidism, *n* (%)		17 (18)	12 (41)	5 (7.5)	0.86 (0.41 to 1.30)
Renal disease, *n* (%)		5 (5.2)	5 (17)	-	0.65 (0.20 to 1.10)
Cardiac disease, *n* (%)		10 (10)	10 (34)	-	1.00 (0.57 to 1.50)
Liver disease, *n* (%)		2 (2.1)	2 (6.9)	-	0.38 (− 0.05 to 0.82)
Previous bilateral adnexectomy, *n* (%)		11 (11)	3 (10)	8 (12)	0.06 (− 0.37 to 0.49)
FMR1 premutation, *n* (%)		4 (4.1)	-	4 (6.0)	
Previous gonadotoxic chemotherapy, *n* (%)		5 (5.2)	-	5 (7.5)	

^a^Primary ovarian insufficiency

^b^Confidence interval

^c^Interquartile range

^d^Hormone replacement treatment

^e^Body mass index

Median age at IVF was similar between women with Turner syndrome and those with other POI (31 [30–32] vs. 32 [30–35] years). Women with Turner syndrome had a higher median BMI compared with other POI patients.

Spontaneous puberty was significantly less frequent among women with Turner syndrome (36% vs. 82%). Accordingly, the duration of HRT prior to IVF was substantially longer in the Turner group, with a median duration of 15.5 years [12.0–17.0], compared with 3.0 years [2.0–6.0] in other POI patients.

Gravidity and parity were low in both groups, with median values of 0 (IQR 0–0), indicating that most patients were nulligravid and nulliparous at the time of inclusion.

Median uterine volume measured prior to IVF did not differ significantly between groups (44 mL [25–51] in Turner vs. 32 mL [21–48] in other POI).

No patients presented with adenomyosis, fibroids, or significant uterine abnormalities likely to affect uterine volume estimation or implantation potential.

Regarding medical history, women with Turner syndrome more frequently presented with hypothyroidism, renal disease, and cardiac disease, whereas the prevalence of diabetes mellitus, liver disease, and previous adnexectomy was comparable between groups.

In this cohort, 10 women with Turner syndrome had underlying cardiac conditions, but none met criteria for contraindication to pregnancy following multidisciplinary evaluation.

All women in the non-Turner POI group had a normal 46,XX karyotype. Among them, four patients had a positive FMR1 premutation, eight had iatrogenic POI following bilateral adnexectomy, and five had iatrogenic POI following chemotherapy. No patients had a history of pelvic radiotherapy. Autoimmune POI was not identified, as all patients tested negative for anti-21-hydroxylase antibodies. The remaining 50 patients were classified as having idiopathic POI.

### Embryo transfer characteristics

Characteristics of endometrial preparation, embryo, and embryo transfer outcomes are presented in Table 2.

**Table 2:** Embryo transfer characteristics

	*N*	Overall	Turner *N* = 30		Other POI^a ^*N* = 67	Difference (G5% CI^b^)
Endometrial preparation characteristics						
Duration of estradiol monotherapy (days), median (IǪR^c^)	209	20 (16–27)	19 (15–23)		21 (17–29)	0.44 (0.15 to 0.72)
Vaginal estradiol 4–6–8 mg, *n* (%)	236	187 (79)	62 (68)		125 (86)	
Vaginal estradiol 8 mg, *n* (%)		14 (5.9)	9 (9.9)		5 (3.4)	0.44 (0.18 to 0.71)
Transdermal patch, *n* (%)		35 (15)	20 (22)		15 (10)	
Vaginal progesterone 600 mg, *n* (%)	242	199 (82)		79 (87)	120 (79)	0.20 (− 0.06 to 0.46)
Vaginal progesterone 600 mg + DYD^d^ 30 mg, *n* (%)		43 (18)		12 (13)	31 (21)	
Endometrial thickness (mm), median (IǪR^c^)	209	9.00 (7.80–10.70)		10.00 (8.98–12.30)	8.40 (7.40–10.10)	− 0.81 (− 1.10 to − 0.51)
Mean uterine artery pulsatility index, median (IǪR^c^)	209	1.76 (1.45–2.12)		1.80 (1.39–2.13)	1.72 (1.46–2.12)	0.01 (− 0.27 to 0.29)
Presence of hydrometra, *n* (%)	208	1 (0.5)		-	1 (0.8)	0.12 (− 0.16 to 0.41)
Embryo characteristics						
Number of transfers per patient, median (IǪR^c^)	245	2.00 (1.00–3.00)		2.00 (1.00–3.00)	2.00 (1.00–3.00)	− 0.33 (− 0.59 to − 0.07)
Fresh embryo transfer, *n* (%)	240	116 (48)		34 (37)	82 (55)	0.36 (0.10 to 0.62)
Warmed embryo transfer, *n* (%)		124 (52)		57 (63)	67 (45)	
One embryo transferred, *n* (%)	241	118 (49)		65 (71)	53 (35)	0.79 (0.52 to 1.10)
Two embryo transferred, *n* (%)		123 (51)		26 (29)	97 (65)	
Embryo 1 developmental stage						
Cleavage-stage embryo (day 2–3), *n* (%)	243	123 (47.7)		30 (23.0)	93 (62.0)	− 0.65 (− 0.92 to − 0.39)
Blastocyst stage embryo (day 5–6), *n* (%)		120 (52.3)		65 (77.0)	55 (38.0)	
Embryo 2 developmental stage						
Cleavage-stage embryo (day 2–3), *n* (%)	124	107 (89.0)		20 (84.3)	84 (90.0)	
Blastocyst stage embryo (day 5–6), *n* (%)		17 (11)		5 (16)	12 (10)	
Donor age (years), median (IǪR^c^)	221	32 (28–34)		31 (27–33)	32 (29–34)	0.29 (0.02 to 0.56)
Embryo transfer outcomes						
No pregnancy, *n* (%)	245	115 (47)		36 (38)	79 (52)	0.37 (0.11 to 0.63)
Live birth, *n* (%)		68 (28)		27 (29)	41 (27)	
Miscarriage, *n* (%)		53 (21.4)		27 (18.6)	26 (17.6)	
Ectopic pregnancy, *n* (%)		5 (2.0)		2 (2.1)	3 (2.0)	
Intrauterine fetal demise, *n* (%)		3 (1.2)		2 (2.1)	1 (0.7)	
Termination of pregnancy, *n* (%)		1 (0.4)		-	1 (0.7)	

^a^Primary ovarian insufficiency

^b^Confidence interval

^c^Interquartile range

^d^Dydrogesterone

The median duration of estradiol monotherapy before progesterone initiation was slightly shorter in women with Turner syndrome (19 [15–23] days) compared with other POI patients (21 [17–29] days). Vaginal estradiol administration was the most frequently used route in both groups, although transdermal estradiol patches were more commonly used in the Turner group.

Endometrial thickness on the day of progesterone initiation was significantly greater in women with Turner syndrome (10.0 mm [8.98–12.3]) compared with other POI patients (8.4 mm [7.4–10.1]). Mean uterine artery pulsatility index and the presence of hydrometra were similar between groups.

The median number of embryo transfers per patient was identical in both groups (2.0 [1.0–3.0]). Fresh embryo transfers were less frequent in women with Turner syndrome, who more often underwent frozen embryo transfers.

Elective single-embryo transfer (eSET) was significantly more frequent in the Turner group (71% vs. 35%), whereas double-embryo transfer (DET) predominated in the other POI group. Blastocyst-stage embryos (day 5–6) were more frequently transferred in women with Turner syndrome for the first embryo transferred.

Median donor age was similar between groups.

Live birth rate per embryo transfer was 28% overall, with comparable rates between women with Turner syndrome (29%) and those with other POI (27%). Rates of miscarriage, ectopic pregnancy, intrauterine fetal demise, and pregnancy termination were low and did not differ significantly between groups.

### Obstetrical and neonatal outcomes

Obstetrical and neonatal outcomes in singleton pregnancies are presented in Table 3.

**Table 3:** Obstetric and neonatal outcomes in singleton pregnancies

	*N*	Overall	Turner *N* = 30	Other POI^a ^*N* = 67	Difference (G5% CI^b^)
Obstetric outcomes					
Hypertensive disorder					
Gestational hypertension, *n* (%)	53	4 (7.5)	2 (7.4)	2 (7.7)	0.36 (− 0.19 to 0.90)
Preeclampsia, *n* (%)		7 (13)	2 (7.4)	5 (19	
Gestational diabetes, *n* (%)	52	8 (15)	5 (19)	3 (12)	0.18 (− 0.36 to 0.73)
Placenta previa, *n* (%)	52	1 (1.9)	1 (3.7)	-	
Hepatic disorder					
Intrahepatic cholestasis of pregnancy, *n* (%)	_51_	1 (2.0)	1 (4.2)	**-**	0.38 (− 0.14 to 0.89)
HELLP^e^ syndrome, *n* (%)		1 (2.0)	**-**	1 (3.7)	
Onset of labor					
Spontaneous labor, *n* (%)	54	14 (26)	6 (22)	8 (30)	0.57 (0.03 to 1.10)
Induction of labor, *n* (%)		21 (39)	8 (30)	13 (48)	
Elective cesarean, *n* (%)		19 (35)	13 (48)	6 (22)	
Mode of delivery					
Spontaneous vaginal delivery, *n* (%)	54	14 (26)	4 (15)	10 (37)	0.77 (0.21 to 1.30)
Vacuum-assisted vaginal delivery, *n* (%)		4 (7.4)	1 (3.7)	3 (11)	
Forceps-assisted vaginal delivery, *n* (%)		5 (9.3)	2 (7.4)	3 (11)	
Spatula-assisted vaginal delivery, *n* (%)		1 (1.9)	1 (3.7)	-	
Cesarean delivery, *n* (%)		30 (56)	19 (70)	11 (41)	
Fetal presentation					
Cephalic, *n* (%)	54	43 (80)	20 (74)	23 (85)	0.50 (− 0.04 to 1.00)
Breech, *n* (%)		8 (15)	4 (15)	4 (15)	
Transverse, *n* (%)		3 (5.6)	3 (11)	-	
Gestational age at delivery					
Term, *n* (%)	54	43 (80)	22 (81)	21 (78)	0.70 (0.15 to 1.20)
Postterm, *n* (%)		4 (7.4)	**-**	4 (15)	
Moderate preterm, *n* (%)		5 (9.3)	4 (15)	1 (3.7)	
Extremely preterm, *n* (%)		2 (3.7)	1 (3.7)	1 (3.7)	
Postpartum hemorrhage, *n* (%)	52	16 (31)	10 (37)	6 (24)	0.29 (− 0.26 to 0.83)
Neonatal outcomes					
Birthweight (grams), median (IǪR^c^)	52	3065 (2630–3360)	2930 (2600–3220)	3090 (2720–3600)	0.22 (− 0.33 to 0.76)
Growth percentile					
10th–90th percentile, *n* (%)	43	35 (81)	19 (90)	16 (73)	0.54 (− 0.07 to 1.20)
> 90th percentile, *n* (%)		2 (4.7)	1 (4.8)	1 (4.5)	
3rd–10th percentile, *n* (%)		-	**-**	**-**	
< 3rd percentile, *n* (%)		6 (14)	1 (4.8)	5 (23)	
5 min Apgar score, *n* (%)	49	10.00 (10.00–10.00)	10.00 (10.00–10.00)	10,00 (10.00–10.00)	0.13 (− 0.39 to 0.66)
Arterial cord pH, *n* (%)	34	7.23 (7.19–7.28)	7.23 (7.20–7.29)	7.23 (7.17–7.28)	0.31 (− 0.37 to 1.00)
Neonatal outcome					
No admission to NICU^d^, *n* (%)	^52^	48 (92)	25 (96)	23 (88)	0.29 (− 0.25 to 0.84)
NICU^d^ admission, *n* (%)		4 (7.7)	1 (3.8)	3 (12)	
Neonatal death, *n* (%)		-	**-**	**-**	

^a^Primary ovarian insufficiency

^b^Confidence interval

^c^Interquartile range

^d^Neonatal intensive care unit

^e^Hemolysis elevated liver enzymes low platelet count

All pregnancies in the Turner group were singleton, whereas twin pregnancies occurred exclusively in the other POI group (*n* = 8, 20%) and were excluded from obstetric and neonatal outcome analyses.

Hypertensive disorders of pregnancy occurred in both groups, including gestational hypertension and preeclampsia. No significant differences were observed between groups regarding most obstetrical complications.

No cardiovascular complications, including aortic dissection, were observed during assisted reproductive treatment or throughout pregnancy in women with Turner syndrome.

Elective cesarean delivery was more frequent in women with Turner syndrome (70% vs 41%).

Rates of non-cephalic presentation and postpartum hemorrhage were comparable between groups, whereas preterm birth appeared slightly more frequent in women with Turner syndrome (Table 3).

Median birthweight of the first infant was similar between groups.

Apgar scores at 5 min and umbilical arterial pH values were comparable. Admission to the neonatal intensive care unit (NICU) was also comparable.

### Live birth according to embryo transfer rank

When live birth rates were analyzed according to embryo transfer rank, a significant difference was observed at the first embryo transfer. Women with Turner syndrome had a significantly lower likelihood of achieving a live birth at the first embryo transfer compared with women with POI of other etiologies (OR 0.09; 95% CI 0.01–0.64; *p* = 0.017).

In contrast, among women undergoing three or more embryo transfer attempts, no statistically significant difference in live birth rates was observed between the two groups (OR 9.78; 95% CI 0.67–14.2; *p* = 0.095) (Table 4).

**Table 4:** Association between Turner syndrome and live birth outcomes: adjusted model

Embryo transfer number	Comparison	OR^a^	G5% CI^b^	*p*-value
1	Yes/No	0.09	(0.01; 0.64)	0.017
3 or more	Yes/No	9.78	(0.67; 14.20)	0.095

Adjusted for BMI, history of spontaneous puberty, type of embryo (fresh or frozen), day of embryo development, number of embryos transferred, type of artificial cycle, duration of estradiol monotherapy, and endometrial thickness prior to embryo transfer

^a^Odds ratio

^b^Confidence interval

### Association between uterine volume and obstetric outcomes

In women with Turner syndrome, decreasing uterine volume was significantly associated with an increased risk of intrauterine growth restriction (OR 1.10, 95% CI 1.04–1.25; *p* = 0.03) and preeclampsia (OR 1.05, 95% CI 1.02–1.11; *p* = 0.013). A similar trend was observed for non-cephalic presentation, although this did not reach statistical significance (OR 1.02, 95% CI 1.00–1.05; *p* = 0.059) (Table 5).

**Table 5:** Uterine volume and risk of obstetric complications

Group	Obstetric complication	OR^a^	G5% CI^b^	*p*-value
Other POI^c^	Intra-uterine growth restriction	1.02	(1.00; 1.05)	0.067
Turner		1.10	(1.04; 1.25)	0.030
Other POI^c^	Preeclampsia	1.01	(0.99; 1.04)	0.210
Turner		1.05	1.02; 1.11)	0.013
Other POI^c^	Non-cephalic presentation	1.04	(1.01; 1.08)	0.021
Turner		1.02	(1.00; 1.05)	0.059
Other POI^c^	Adverse neonatal outcome	1.10	(1.04; 1.27)	0.031
Turner		1.09	(1.03; 1.21)	0.022

^a^Odds ratio

^b^Confidence interval

^c^Primary ovarian insufficiency

In women with POI of other etiologies, lower uterine volume was associated with a higher risk of non-cephalic fetal presentation (OR 1.04, 95% CI 1.01–1.08; *p* = 0.021) and adverse neonatal outcomes (OR 1.10, 95% CI 1.04–1.27; *p* = 0.031). A non-significant trend toward increased risk of intrauterine growth restriction was observed (OR 1.02, 95% CI 1.00–1.05; *p* = 0.067). No significant association was found between uterine volume and preeclampsia (OR 1.01, 95% CI 0.99–1.04; *p* = 0.20).

### Association between duration of hormone replacement therapy and uterine volume

The association between the duration of prior HRT and uterine volume was evaluated separately in women with Turner syndrome and in those with premature ovarian insufficiency of other etiologies.

In women with POI of other etiologies, uterine volume increased by an estimated 0.04 mL per year of HRT (95% CI − 0.13 to 0.22; *p* = 0.627).

In women with Turner syndrome, the estimated increase in uterine volume was 0.02 mL per year of HRT (95% CI − 0.10 to 0.14; *p* = 0.752).

In both groups, the association between HRT duration and uterine volume was not statistically significant, and the magnitude of the effect was very small (Table 6).

**Table 6:** Relationship between uterine volume and duration of prior HRT^a^

Group	Increase in uterine volume (mL) per year of HRT^a^	G5% CI^b^	*p*-value
Other POI^c^	0.04	(− 0.13; 0.22)	0.627
Turner	0.02	(− 0.10; 0.14)	0.752

^a^Hormone replacement therapy

^b^Confidence interval

^c^Primary ovarian insufficiency

## Discussion

### Principal findings

In this retrospective cohort study comparing women with Turner syndrome and women with premature ovarian insufficiency of other etiologies undergoing IVF with oocyte donation, several important findings emerge. First, live birth rates per embryo transfer were similar between groups, confirming that oocyte donation is an effective reproductive option for women with Turner syndrome. However, women with Turner syndrome required a higher number of embryo transfer attempts to achieve a live birth, with a significantly lower probability of success at the first attempt; this difference was no longer observed after three or more transfers.

Second, lower pre-treatment uterine volume was associated with an increased risk of obstetric complications, including hypertensive disorders of pregnancy, intrauterine growth restriction, non-cephalic presentation, and adverse neonatal outcomes. Importantly, this association was observed both in women with Turner syndrome and in those with other causes of POI.

Third, uterine volume was not significantly associated with the duration of prior HRT in either group. The estimated annual increase in uterine volume was minimal and not statistically significant, suggesting that the relationship between HRT duration and uterine development cannot be reliably established in this study.

### Live birth outcomes in Turner syndrome versus other POI

In the present study, women with Turner syndrome required more embryo transfer attempts to achieve a live birth than women with POI of other etiologies.

One intriguing hypothesis to explain the lower likelihood of live birth at the first embryo transfer in women with Turner syndrome may relate to the interaction between long-term estrogen exposure and short-term high-dose estrogen regimens used for endometrial preparation prior to transfer. In clinical practice, HRT in women with Turner syndrome for pubertal induction or adult maintenance typically involves physiologic, low-to-moderate doses of estrogen titrated gradually over years to mimic normal puberty and support systemic health [12]. These conventional adult replacement regimens, as recommended in clinical guidelines, involve doses of transdermal 17-β estradiol around 50–200 µg/day or oral estradiol 2–4 mg/day [13]. In contrast, protocols for endometrial preparation in IVF in our study employ higher doses of estradiol up to 8 mg daily to achieve optimal endometrial thickness for implantation, a level that exceeds typical replacement dosing.

This discrepancy raises the possibility that a period of “priming” the uterus with higher doses of estrogen prior to any embryo transfer—potentially in one or two cycles without transfer (“dummy” HRT cycles)—might enhance uterine responsiveness and improve first transfer outcomes. This hypothesis is supported by the study by Khastgir et al., which reported higher pregnancy rates in subsequent embryo transfers compared with the first transfer [14]. Higher estradiol doses in HRT are associated with greater endometrial thickness at embryo transfer [15].

To our knowledge, no prospective studies have systematically tested the effect of high-dose estrogen priming cycles prior to embryo transfer in either the general oocyte donation IVF population or in women with Turner syndrome. However, the widely different dosing strategies between chronic HRT in Turner syndrome and acute estrogen-driven endometrial preparation protocols suggest a fertile ground for future research. Well-designed randomized controlled trials or observational studies examining whether pre-transfer hormonal priming improves implantation and live birth rates in this high-risk population could have important clinical implications.

Previous studies have generally reported similar clinical pregnancy and live birth rates after oocyte donation in women with Turner syndrome compared with other women with ovarian insufficiency and normal karyotypes [16]. However, several authors have noted an increased risk of early pregnancy loss in Turner syndrome, which may contribute to the need for repeated embryo transfers before achieving a successful outcome [3]. Our results are consistent with this observation.

Notably, the live birth rate per embryo transfer observed in women with Turner syndrome in our cohort was higher than that reported in many previous series. In our study, the live birth rate per transfer in Turner syndrome was approximately 29%, compared with an estimated 17% per embryo transfer reported in a recent 2024 meta-analysis [17].

The observed difference in live birth rates may be explained by the modalities of HRT used for endometrial preparation. Women with Turner syndrome are known to exhibit suboptimal endometrial growth under estrogen stimulation [18], which can be improved by higher estrogen doses [19]. However, estrogen should not be administered as monotherapy for more than 28 days [20] and should probably be prescribed in a uterus that has been previously estrogen-primed. Indeed, initiation of estrogen therapy after a prolonged hypoestrogenic period may induce hydrometra due to the resumption of endometrial glandular activity, thereby impairing embryo implantation [21]. In our study, no embryo transfer was performed when endometrial thickness was below 7 mm, a threshold below which implantation rates are known to decline [22]. Median durations of estradiol monotherapy were 19 and 21 days in women with Turner syndrome and in women with POI of other etiologies, respectively, resulting in median endometrial thicknesses of 10.0 mm and 8.4 mm. High-dose estrogen was administered via the vaginal route in 85% of the patients in the cohort.

Progesterone plays a pivotal role in endometrial receptivity and is essential for preventing early pregnancy loss [23]. Its administration is therefore mandatory in this clinical context. In our study, all patients received vaginal micronized progesterone at a dose of 600 mg daily, allowing for a uterine first-pass effect that enhances endometrial progesterone exposure [24]. In addition, 18% of patients received combined luteal phase support with vaginal micronized progesterone (600 mg daily) and oral dydrogesterone (30 mg daily). This combined regimen has been suggested to improve live birth rates compared with vaginal progesterone alone, according to a study [25].

In addition, women with Turner syndrome in Lyon undergo rigorous multidisciplinary assessment, particularly cardiovascular screening, prior to pregnancy [9]. This selection process may contribute to improve transfer outcomes while potentially excluding the most severe phenotypes from attempting pregnancy. Beyond this selection effect, the French healthcare system enables coordinated, lifelong follow-up of patients with Turner syndrome, often beginning in childhood. As a result, many patients benefit from early, continuous, and appropriately dosed HRT, which may promote more optimal uterine development and endometrial responsiveness by the time reproductive treatment is initiated.

### Uterine volume and response to hormone replacement therapy

In our cohort, uterine volume prior to IVF did not differ significantly between women with Turner syndrome and those with POI of other etiologies. We did not observe a significant association between uterine volume and duration of HRT in either group. Several studies have shown that even after appropriately dosed estrogen replacement during induced puberty, uterine volume in girls with Turner syndrome remains significantly smaller than in peers without ovarian insufficiency, suggesting an intrinsic limitation of uterine growth that is not fully corrected by HRT [26].

These data support the hypothesis that uterine development in Turner syndrome is characterized by a blunted response to estrogen, potentially related to absent spontaneous puberty, altered developmental programming, or X-chromosome haploinsufficiency, and that long-term hormone replacement may partially compensate for this reduced sensitivity.

Other studies have suggested that appropriately dosed and prolonged HRT may allow uterine growth in women with Turner syndrome to reach volumes considered compatible with pregnancy [27, 28]. These findings suggest that estrogen therapy promotes substantial uterine growth in Turner syndrome but may not fully restore normal uterine development.

Our results are consistent with this nuanced view. Although uterine volume prior to IVF did not differ significantly between women with Turner syndrome and those with POI of other etiologies, Turner patients had been exposed to estrogen replacement for a substantially longer duration. This suggests that comparable uterine volumes may reflect prolonged cumulative estrogen exposure rather than equivalent uterine sensitivity or physiology.

Importantly, the longer duration of HRT observed in women with Turner syndrome in our study should be interpreted in light of differences in pubertal development between groups. In our cohort, spontaneous puberty was significantly less frequent in women with Turner syndrome compared with those with POI of other etiologies, leading to earlier initiation of hormone replacement therapy for pubertal induction and, consequently, a longer cumulative duration of estrogen exposure prior to ART. In contrast, most women with other causes of POI experienced spontaneous puberty, and HRT was therefore initiated later, typically after the onset of ovarian insufficiency. Thus, differences in HRT duration between groups likely reflect distinct clinical trajectories rather than differences in treatment strategies alone. This prolonged exposure to estrogen in women with Turner syndrome may partly explain the absence of a significant difference in uterine volume at the time of IVF.

Importantly, the analysis of uterine volume in relation to HRT duration was conducted at a population level using cross-sectional data, rather than longitudinal individual follow-up. Therefore, the estimated association reflects an average relationship between duration of HRT and uterine volume and does not represent true intra-individual uterine growth over time. This limitation may partly explain the absence of a significant association and limits causal interpretation.

In addition, the route of estrogen administration may also influence uterine development. Transdermal estradiol has been shown to provide more physiological hormone levels and may be associated with improved uterine growth compared with oral formulations, particularly during pubertal induction [28]. Differences in the type and route of estrogen therapy prior to ART, which were not fully controlled for in this study, may therefore have contributed to interindividual variability in uterine volume and should be considered when interpreting our findings.

Several mechanisms may underlie a reduced uterine sensitivity to estrogen in Turner syndrome. The absence of spontaneous puberty and menarche in most patients deprives the uterus of a critical developmental window during which endogenous estrogen induces structural, vascular, and myometrial maturation that may not be fully replicated by exogenous hormones initiated later or delivered in non-physiological patterns [29]. In addition, X-chromosome haploinsufficiency may contribute to altered estrogen signaling, as several genes involved in uterine development and endometrial receptivity escape X-inactivation and may be underexpressed in Turner syndrome, potentially leading to a blunted tissue-level response to estrogen despite adequate circulating hormone levels [30].

From a clinical perspective, these findings support the systematic assessment of uterine volume in women with POI undergoing assisted reproductive treatment, particularly in those with Turner syndrome. They also reinforce the importance of early, sustained, and adequately dosed HRT, ideally initiated during adolescence, to promote optimal uterine development. Although the absolute annual increase in uterine volume associated with HRT was modest, its cumulative effect over many years may be clinically meaningful, especially given the association between decreasing uterine volume and adverse obstetric outcomes observed in our study.

### Uterine volume and obstetric outcomes

It is well-established that pregnancies conceived via ART, such as IVF, are associated with increased maternal and perinatal morbidity compared with naturally conceived pregnancies [31]. Moreover, the risk of severe acute maternal morbidity has been shown to be higher among women undergoing oocyte donation compared with conventional IVF using autologous oocytes [8].

In women with Turner syndrome, these risks are compounded, with studies reporting elevated rates of obstetric complications such as cesarean delivery and small-for-gestational-age infants, underscoring the high-risk nature of these pregnancies [32], also for spontaneous pregnancies [33, 34].

In this study, we demonstrated that decreasing pre-IVF uterine volume seems associated with an increased risk of adverse obstetric and neonatal outcomes, particularly in women with Turner syndrome. To minimize confounding, analyses were restricted to singleton pregnancies, excluding twin gestations, which are known to carry an intrinsic excess risk of obstetric morbidity [35]. Uterine volume was categorized using the median value and analyzed separately in Turner and non-Turner POI populations.

In women with Turner syndrome, decreasing uterine volume was significantly associated with intrauterine growth restriction, preeclampsia, and adverse neonatal outcomes. A trend toward an increased risk of non-cephalic presentation was also observed. These associations were either weaker or not statistically significant in women with POI of other etiologies, although similar directions of effect were noted for some outcomes. Notably, the magnitude of the associations was modest, but consistent across several clinically relevant endpoints, supporting a potential link between preconception uterine development and subsequent placental and fetal outcomes.

The relationship between uterine size and obstetric complications may reflect impaired placentation and uteroplacental perfusion in a structurally or functionally underdeveloped uterus. Reduced uterine volume may be associated with altered myometrial architecture, decreased uterine compliance, and impaired spiral artery remodeling, all of which are key determinants of placental development and fetal growth [36]. These mechanisms are consistent with the observed associations with fetal growth restriction and hypertensive disorders of pregnancy, particularly in women with Turner syndrome, in whom a significant association with preeclampsia was observed, whereas no such association was found in women with POI of other etiologies.

Non-cephalic presentation was also more frequent in women with decreasing uterine volumes, particularly in the non-Turner POI group, with a similar trend observed in Turner syndrome. A reduced uterine cavity size or altered uterine shape may mechanically limit fetal mobility and favor malpresentation, as previously suggested in other contexts of congenital uterine anomalies [37].

From a clinical standpoint, these findings suggest that preconception uterine volume assessment may help identify women with POI—particularly those with Turner syndrome—at higher risk of obstetric and neonatal complications following IVF with oocyte donation. While uterine volume alone should not be used as a contraindication to pregnancy, it may represent a useful marker for risk stratification, counseling, and tailored antenatal surveillance in this high-risk population.

### Strengths and limitations

The strengths of this study include the relatively large number of embryo transfers analyzed, the long study period, and the detailed characterization of reproductive, hormonal, uterine, obstetric, and neonatal parameters in a well-defined population of women with premature ovarian insufficiency undergoing IVF with oocyte donation. To our knowledge, this is one of the few studies to specifically explore the relationship between pre-IVF uterine volume, prior HRT, and obstetric outcomes in women with Turner syndrome compared with other POI etiologies. Importantly, obstetric analyses were restricted to singleton pregnancies, thereby limiting confounding related to the intrinsic morbidity of multiple gestations.

Several limitations should nevertheless be acknowledged. First, the retrospective design exposes the study to potential selection bias and missing data, inherent to observational research. Second, the number of women with Turner syndrome remains limited, which may have reduced statistical power to detect small but clinically relevant differences, particularly for secondary outcomes. However, Turner syndrome is a rare condition, and our cohort is comparable in size to those reported in the literature.

Third, uterine volume was assessed using two-dimensional transvaginal ultrasound measurements and calculated using an ellipsoid formula (L × W × AP × 0.52) [11]. While this method is widely used in clinical practice, there is no clear consensus regarding the optimal formula for uterine volume calculation, and measurement variability cannot be excluded [38]. In addition, uterine volume represents a static anatomical parameter and may not fully capture functional aspects of uterine receptivity or vascular adaptation, which could be better assessed using advanced imaging techniques such as three-dimensional ultrasound or Doppler flow analysis.

Fourth, the imbalance in the proportion of fresh and frozen embryo transfers between groups represents a potential limitation, as these approaches may be associated with different implantation and live birth rates. Although this factor was adjusted for in the statistical models, residual confounding cannot be excluded. Importantly, none of the embryos in this cohort underwent PGT-A, in accordance with French regulations, thereby limiting potential differences in embryo selection between fresh and frozen cycles.

Differences in the proportion of single versus double embryo transfers between groups may also have influenced reproductive outcomes. Although this factor was included in the adjusted models, residual confounding cannot be excluded.

Fifth, although we explored the association between uterine volume and obstetric outcomes, causality cannot be inferred. Decreasing uterine volume may be a marker of more global uterine or vascular immaturity rather than a direct determinant of adverse pregnancy outcomes. Finally, despite adjustment strategies and stratified analyses, residual confounding—particularly related to cardiovascular status, autoimmune conditions, or subtle differences in hormonal exposure—cannot be entirely excluded, especially in women with Turner syndrome.

Finally, the relationship between HRT duration and uterine volume was assessed using cross-sectional data rather than longitudinal measurements, which limits the ability to accurately estimate uterine growth over time. In addition, differences in HRT duration between groups may introduce bias, making comparisons between Turner syndrome and other POI etiologies difficult to interpret.

### Clinical implications

Our findings have several important clinical implications.

First, they support the effectiveness of IVF with oocyte donation in women with Turner syndrome, with live birth rates per embryo transfer comparable to those observed in other POI etiologies, albeit at the cost of a greater number of attempts.

Second, they highlight the potential importance of early and prolonged HRT to promote uterine development, particularly in women with absent spontaneous puberty.

Third, the association between decreasing pre-IVF uterine volume and adverse obstetric and neonatal outcomes suggests that uterine volume assessment could contribute to preconception risk stratification and individualized counseling. In women with Turner syndrome and reduced uterine volume, closer antenatal surveillance may be warranted, particularly regarding placental function, fetal growth, and hypertensive disorders of pregnancy.

## Data Availability

The datasets generated and analyzed during the current study are available from the corresponding author on reasonable request.
